# Ancient Schwannoma of Ansa Cervicalis: A Rare Clinical Entity and Review of the Literature

**DOI:** 10.1155/2015/578467

**Published:** 2015-10-08

**Authors:** Satyajit Rath, Prakash K. Sasmal, Kaushik Saha, N. Deep, Pritinanda Mishra, Tushar S. Mishra, Rakesh Sharma

**Affiliations:** ^1^Department of General Surgery, All India Institute of Medical Sciences, Bhubaneswar, Odisha, India; ^2^Department of Pathology, All India Institute of Medical Sciences, Bhubaneswar, Odisha, India; ^3^Department of Radio-Diagnosis, All India Institute of Medical Sciences, Bhubaneswar, Odisha, India

## Abstract

Ancient schwannoma is an uncommon variant of schwannoma, a benign tumor arising from the nerve sheath. It is reported to arise from any nerves except optic and olfactory. However, only six cases of ancient schwannomas arising from ansa cervicalis nerve have been reported to date in English literature. Proper preoperative evaluation is necessary to rule out other causes of neck mass such as thyroid lesions, lymphadenopathy, and carotid body tumor. We report a case of ancient schwannoma arising from the ansa cervicalis nerve. The origin of the lesion from ansa cervicalis was confirmed by intraoperative finding. Postoperative histopathological examination revealed degenerative changes including pleomorphism, cellular atypia, large nuclei with prominent nucleoli, and paucity of mitotic figures. Periphery of the mass showed nuclear palisading with characteristic verocay bodies. Immunohistochemical evaluation for S-100 showed diffuse positivity of the tumor cells, thereby confirming the diagnosis of schwannoma. We consider that schwannoma of cervical region can have origin from any nerve and should try to identify the origin pre- and intraoperatively. The postoperative complications depend on the nerve of origin and the precision of the surgery performed.

## 1. Introduction

Schwannomas are benign tumors arising from nerve sheath, also named as neuromas, neurilemmomas, or neurinomas [1, 2]. Ancient schwannoma is an uncommon variant of schwannoma which is very slow growing neoplasm. The term “ancient” was proposed to describe a group of neural tumors showing degenerative changes, diffuse hypocellular areas, nuclear hyperchromasia, and marked nuclear atypia [3]. Schwannoma can arise from all types of nerves except the optic and the olfactory nerves, but schwannoma of the ansa cervicalis is extremely rare and is reported in only six cases to date. Because of the rarity of the ansa cervicalis schwannoma, it is seldom considered in the differential diagnosis of schwannoma of the cervical region [1, 2, 4]. The schwannomas arising in the head and neck region mostly originate from the vagus nerve or from the sympathetic nervous system [5]. They usually manifest as a painless mass in the neck with an indolent course and varying pressure symptoms. Malignant transformation is rarely seen, but is often erroneously suspected so, due to its presentation with atypical features [6, 7]. Preoperative investigations are necessary to rule out the frequent causes of neck masses, like thyroid malignancy, lymphadenopathy, or paraganglioma. Surgical excision of the tumor with utmost preservation of the neural function is the accepted treatment for this rare entity [1, 5].

## 2. Case Report

A 62-year-old man presented to the surgical department, with a two-year history of asymptomatic swelling in the right side of neck. The swelling was insidious onset, slowly growing, and was not associated with pain or fever. There was no history of any period of rapid growth, cough, or dysphagia. On examination of the swelling, it was a globular, nontender swelling with a diameter of five centimeters, located in the submandibular triangle. It was not fixed to the overlying skin and was partially mobile. The swelling was firm in consistency with pulsations felt, transmitting from adjoining carotid artery. The swelling was clinically suspected to be a cervical lymph node. Other systemic examinations including laboratory investigations were uninformative.

Ultrasonography of the neck revealed the swelling to be of heterogeneous echo texture and increased vascularity, besides which a few nonsignificant lymph nodes were present in the vicinity of the swelling. The swelling had displaced the internal carotid artery and internal jugular vein anteriorly. A fine needle aspiration (FNA) was performed which revealed spindle cell neoplasm. Contrast enhanced commuted tomography (CECT) showed a heterogeneously enhancing mass with cystic change marked on contrast enhancement, with anterior displacement of the carotid artery and the internal jugular vein.

**Figure 1:**
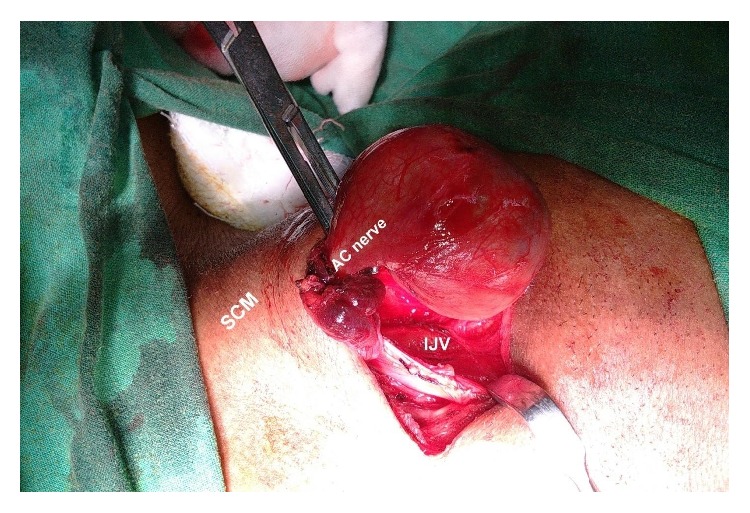
Tumor arising from inferior root of ansa cervicalis nerve.

The patient was operated through a right submandibular skin crease incision. The tumor was laying posterolateral to the internal jugular vein (Figure 1). The vagus, hypoglossal, spinal accessory, glossopharyngeal, and lingual nerves were free from the tumor and were identified and preserved. The overlying lymph nodes were dissected and sent for the histopathological examination separately. The tumor was arising from the inferior root of the ansa cervicalis nerve. The nerve identification was done by stimulation and by noticing the contraction of the strap muscles. The tumor was resected along with a small part of the nerve and sent for the histopathological examination. Postoperative period was uneventful. Patient had no signs or symptoms suggestive of Horner's syndrome, including difficulty in breathing, phonation, or swallowing. Postoperative ophthalmological evaluation was unremarkable. The patient was discharged from the hospital on fifth postoperative day.

**Figure 2:**
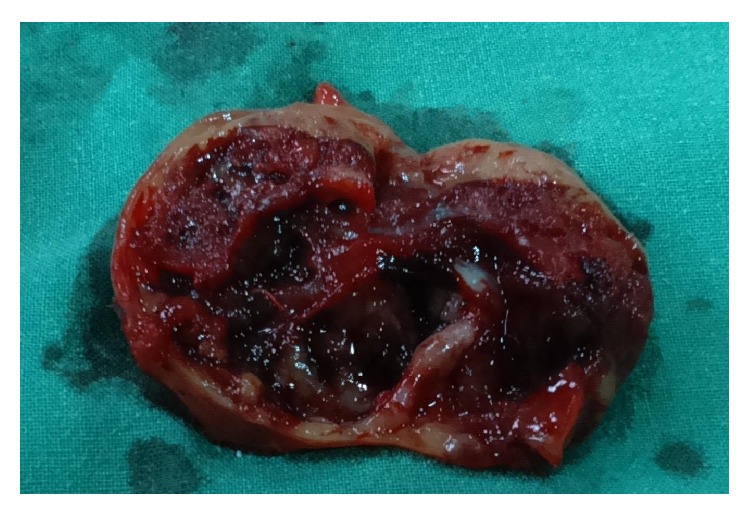
Cut section of the tumor showing solid and cystic areas.

Grossly the specimen was globular in shape, encapsulated, and greyish brown in color, with measurement of 4.5 × 3 × 2.5 cm. The cut section was variegated, solid-cystic with areas of cystic haemorrhagic changes along with foci having soft mucoid consistency (Figure 2).

**Figure 3:**
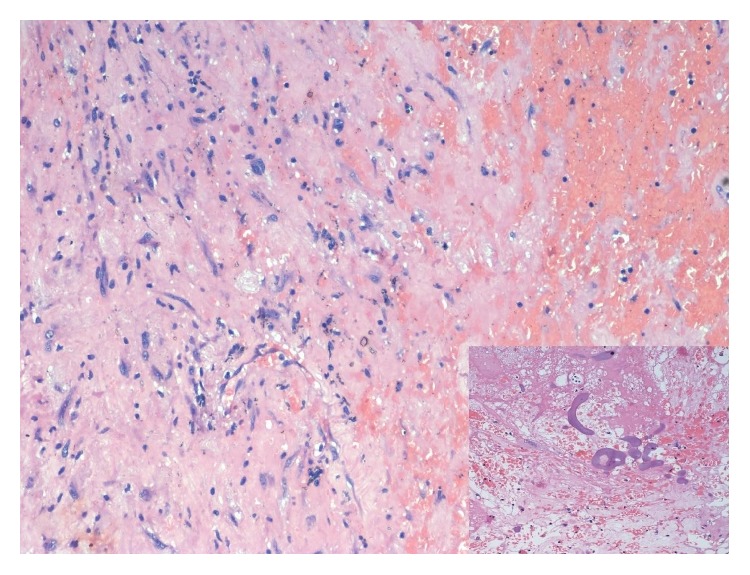
Photomicrograph showing spindle cell neoplasm with cellular atypia and stromal changes (H&E 200x) (inset showing calcification).

**Figure 4:**
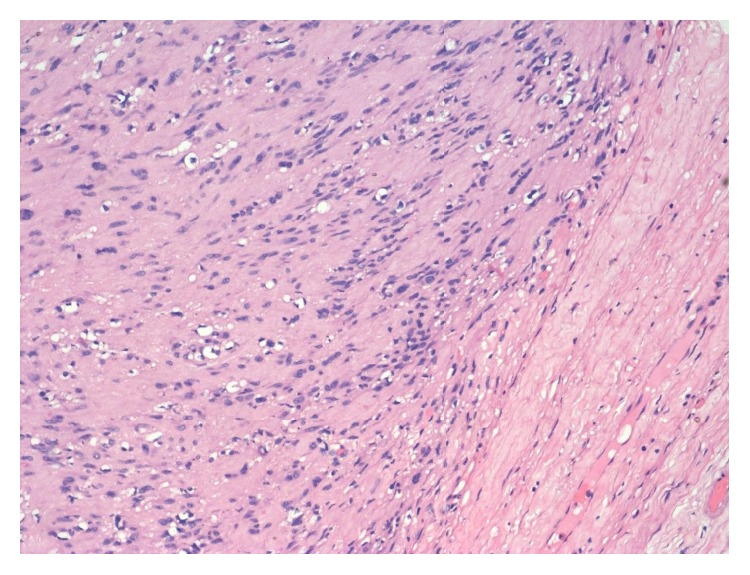
Photomicrograph showing characteristic verocay body with nuclear palisading around fibrillar cellular processes (H&E 200x).

**Figure 5:**
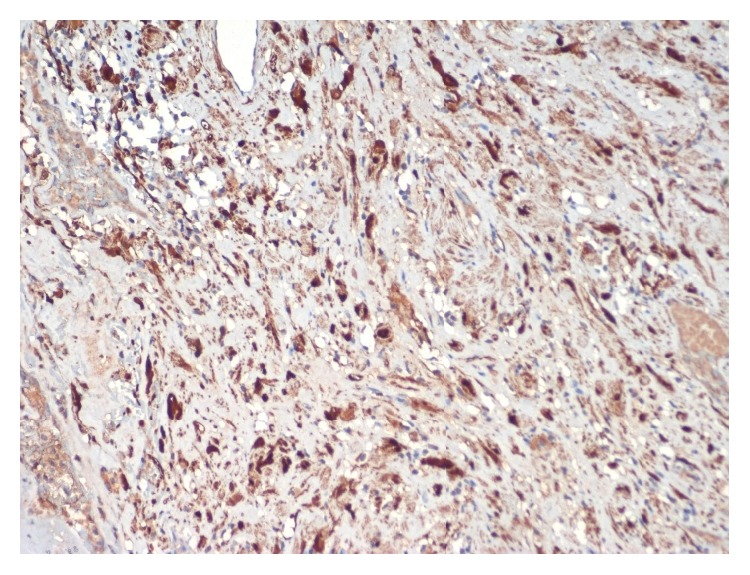
Photomicrograph showing diffuse immunohistochemical positivity for S-100 (HRP 200x).

Microscopically, it showed a well encapsulated tumor composed of cellular and hypocellular areas with compressed neural fascicles at the periphery. The tumor is composed of fusiform cells with elongated fibrillary cytoplasm and wavy buckled nuclei. Degenerative changes were found in a good proportion of cells including pleomorphism, cellular atypia, large nuclei with prominent nucleoli, and paucity of mitotic figures (Figure 3). Central part of the tumor showed vascular proliferation of varying sizes with perivascular hyalinization. Periphery of the mass showed nuclear palisading with characteristic verocay bodies (Figure 4). There were interspersed areas with stromal haemorrhage, desmoplasia, and calcification. With the above findings, the diagnosis of ancient schwannoma was made. Immunohistochemical evaluation for S-100 showed diffuse positivity of the tumor cells, thereby confirming the diagnosis of schwannoma (Figure 5).

## 3. Discussion

Ancient schwannoma of ansa cervicalis is a rare disease and only a few cases had been reported till date. Park et al. reported a case of schwannoma of ansa cervicalis, but the origin of the schwannoma from ansa cervicalis is confirmed intraoperatively by the contraction of the strap muscles on stimulation [6]. de Diego Sastre et al. reported schwannoma originating from ansa cervicalis but the preoperative diagnosis was tumor, arising from thyroid gland [7]. Preoperative diagnosis of ancient schwannoma is difficult, but fine needle aspiration cytology helps in differentiating schwannoma from sarcoma and other differential diagnosis [8]. Clinically these tumors are often mistaken as tumors of the thyroid, enlarged lymph nodes, paraganglioma, or brachial cyst [9, 10]. Schwannoma can have its origin from any nerves except the olfactory and optic nerve which lack the nerve sheath. Ancient schwannoma usually occurs in the head and neck, thorax, retroperitoneum, pelvis, and extremities [11]. They mainly occur in the middle and elderly age, with no sexual predominance. They are usually asymptomatic to start with, but the patient may develop pressure symptoms based on the location of the tumor. It is important to preoperatively rule out carotid body tumor from ancient schwannoma of ansa cervicalis, as the management of both the cases varies.

Contrast enhanced CT scan and MRI are often helpful in diagnosis, but exact origin of the schwannoma is difficult to know preoperatively [12]. The ancient schwannomas appear as well defined homogenous masses which shows cystic changes with contrast enhancement [13], but in our case it was a heterogeneous lesion. Magnetic resonance imaging of a schwannoma usually reveals homogenous low signal intensity on T1-weighted images, while the lesion appears hyperintense in T2-weighted images. We had not done MRI in our patient as it was not economical and would not have changed the overall management.

Ancient schwannoma, also known as degenerative neurilemmoma, is characterized by degeneration and proportionate increase in hypocellular areas (Antoni B). The changes include increased deposition of matrix, perivascular hyalinization, ectatic vessels with thrombus within them, cystic degeneration, and cellular atypia with paucity of mitosis. The changes are attributed to the long duration of schwannoma during its evolution. The predominance of Antoni B area over Antoni A area is radiologically correlated with increased contrast uptake in CECT. As these lesions have diffuse hypocellularity they are difficult to diagnose by fine needle aspiration cytology [14].

Complete surgical excision of the tumor is the treatment of choice, because these are radio-resistant tumors. During excision of the tumor, all efforts should be made to preserve the nerve but it is difficult because of its dense attachment to the tumor. Surgeon needs to be very careful during the operation, as the vagus nerve may be mistaken to be ansa cervicalis. Incomplete excision may result in slow recurrence over months to years [15]. Recurrence and malignant change of ancient schwannoma are very rare but some cases have been reported [16]. The diagnosis is confirmed after histopathological examination, which shows the Antoni type A and type B areas. Tumor cells are usually positive for S-100 antigen in immunohistochemical examination [1].

## 4. Conclusion

Schwannoma can arise from all types of nerves, but origin from the ansa cervicalis is a rare occurrence. It is difficult to diagnose ansa cervicalis as the nerve of origin preoperatively. So to conclude, surgeons should consider that schwannoma of cervical region can have origin from any nerve and should try to identify the origin pre- and intraoperatively. The postoperative complications depend on the nerve of origin and the precision of the surgery performed. This entity may be considered as the differential diagnosis of cervical lymphadenopathy.
